# Molecular docking analysis of MCL-1 inhibitors for breast cancer management

**DOI:** 10.6026/97320630019707

**Published:** 2023-06-30

**Authors:** Alzahrani Abdulrahman, Kamal Mohammad Azhar, Akber Asif Hussain, Asiri Ali Abdullah, Ibrahim Shafei Marui, Ghawi Mohammed Hadi, Alotaibi Hanan Mamdouh, Alam Qamre

**Affiliations:** Department of Applied Medical Sciences, Applied College, Al-Baha University, Al-baha City, Kingdom of Saudi Arabia; Department of Pharmaceutics, College of Pharmacy, Prince Sattam Bin Abdulaziz University, Alkharj 11942, Saudi Arabia; Central Military Laboratory and Blood Bank Department - Virology Division, Prince Sultan Military Medical City, Riyadh 12233, Saudi Arabia; Central Military Laboratory and Blood Bank Department - Microbiology Lab Division, Prince Sultan Military Medical City, Riyadh 12233, Saudi Arabia; Central Military Laboratory and Blood Bank Department - Hematology Lab Division, Prince Sultan Military Medical City, Riyadh 12233, Saudi Arabia; Central Military Laboratory and Blood Bank Department - Hematology Lab Division, Prince Sultan Military Medical City, Riyadh 12233, Saudi Arabia; Central Military Laboratory and Blood Bank Department - Microbiology Lab Division, Prince Sultan Military Medical City, Riyadh 12233, Saudi Arabia; Molecular Genomics and Precision Medicine Department, ExpressMed Diagnostics and Research, Block, 359, Zinj, Kingdom of Bahrain

**Keywords:** Myeloid leukemia 1, breast cancer, virtual screening, apoptosis

## Abstract

Myeloid leukemia 1 (MCL-1), a BCL-2 protein family member, acts as an anti-apoptotic protein by interacting with pro-apoptotic BCL-2 proteins. Its overexpression is frequently observed in numerous cancer types including breast cancer, and is closely linked to the initiation and progression of tumors as well as poor prognosis and resistance to therapeutic interventions. Here, a database of 3402 chemicals with established therapeutic activity against various diseases was chosen and systematically screened against the MCL-1 protein. Visual inspection and binding energy analysis revealed that the compounds OSU-03012, Raltitrexed, Ostarine (MK-2866), Dovitinib (TKI-258), and Varespladib (LY315920) had strong binding affinity for the MCL-1 protein. Notably, their binding affinity was higher than that of the control compounds. These compounds exhibited strong interactions with critical amino acid residues of the MCL-1 protein. Furthermore, these compounds shared several common amino acid residue interactions with the control compounds. These findings suggest that these compounds may be useful as MCL-1 inhibitors in the treatment of breast cancer. However, additional experimental validation is required to confirm these findings.

## Background:

Breast cancer (BC) is currently recognized as one of the most commonly diagnosed malignancies in the worldwide, and it is the fifth leading cause of cancer-related deaths. According to data from GLOBOCAN 2020, approximately 2.3 million new cases of breast cancer are expected to be diagnosed worldwide [1]. In addition to its high prevalence, breast cancer is the leading cause of cancer-related mortality among women globally, accounting for 684,996 deaths at an age-adjusted rate of 13.6 per 100,000 people. Notably, while the incidence rates were highest in developed regions, Asian and African countries accounted for 63% of total breast cancer deaths in 2020 [2]. Myeloid leukemia 1 (MCL-1), BCL-2 protein family member has antiapoptotic properties. It acts by preventing mitochondrial outer membrane permeabilization and the subsequent cytochrome C release from the mitochondria. MCL-1 has gained prominence in the context of BC, with increased levels of MCL-1 protein in primary BC samples consistently associated with a poor patient prognosis [3,4, 5, 6]. There is substantial evidence that MCL-1 targeting is a promising therapeutic avenue in BC. BC cells may rely on MCL-1 for survival in preclinical models, and inhibiting MCL-1 can improve the efficacy of conventional cancer treatments [7,8]. Drug repurposing, also known as drug repositioning, is the process of looking into new applications for existing approved drugs that go beyond their original indications. It provides a promising strategy for expanding the arsenal of cancer treatments and has numerous advantages over developing new drugs from the ground up [9]. Extensive studies are not required because repurposed drugs already have well-characterized pharmacokinetic and pharmacodynamic profiles. This simplifies the translational process, lowers associated costs, and contributes to higher drug development success rates [10]. Due to its reduced risk, expanded therapeutic options, increased revenue potential, and improved patient outcomes, drug repurposing is very important. Examples include antidepressant medications like bupropion and Dapoxetine, which have been successful in treating non-neurological indications like premature ejaculation and quitting smoking, respectively. Drugs like Duloxetine for stress urinary incontinence, Fluoxetine for premenstrual dysphoria, and the non-psychoactive medications Propecia and Minoxidil for hair loss have all been developed as a result of repurposing [11]. Thalidomide is currently used for treating multiple myeloma, demonstrating the success of repurposing in oncology. The ongoing phase II clinical trial for the effective treatment of radiation dermatitis with esomeprazole highlights the possibility of repurposing currently available medications [12]. There are various advantages to drug repurposing over de novo drug development, including greatly decreased time and expense for obtaining approval for a new indication. Repurposed drugs with a track record of safety can be approved in 3-10 years, compared to 10-17 years for novel drugs [13]. Furthermore, repurposed candidates had a better approval rate, with 25% progressing from Phase II to approval, compared to only 10% of new drugs [14]. Using an in-silico approach, this study aimed to find novel MCL-1 inhibitor to fight the BC.

## Methods:

## Retrieval and preparation of target protein:

The 3D structure MCL-1 (PDB ID: 5FDO) was retrieved from the Protein Data Bank [15]. The co-crystal ligand, other heteroatoms, and water molecules were removed from the structure. Subsequently, the protein was modeled using the SWISS-MODEL web tool due to its distorted conformation.

## Compound library preparation:

We selected a database consisting of 3402 preclinical and clinical chemicals known for their activity in treating various diseases, including oncology, cardiology, anti-inflammatory, immunology, neuropsychiatry, analgesia, and others. These chemicals exhibit diverse structural properties, possess medicinal activity, and demonstrate cell permeability.

##  Virtual screening:

The drug discovery process is divided into different stages, including target selection, lead optimization, and preclinical/clinical trials. Computational modeling is strongly related with hit discovery and lead optimization, particularly through the application of structure-based virtual screening (VS) [16]. Docking, the primary computational technique used in VS, has been extensively investigated and widely used in drug discovery during the last decade [17]. The PyRx0.8 tool [18] was used in this study to screen the prepared compound library against the MCL-1 protein. The grid center coordinates were set as X = 7.53, Y = 25.37, and Z = -7.84.

## Results and Discussion:

**Figure 1:**
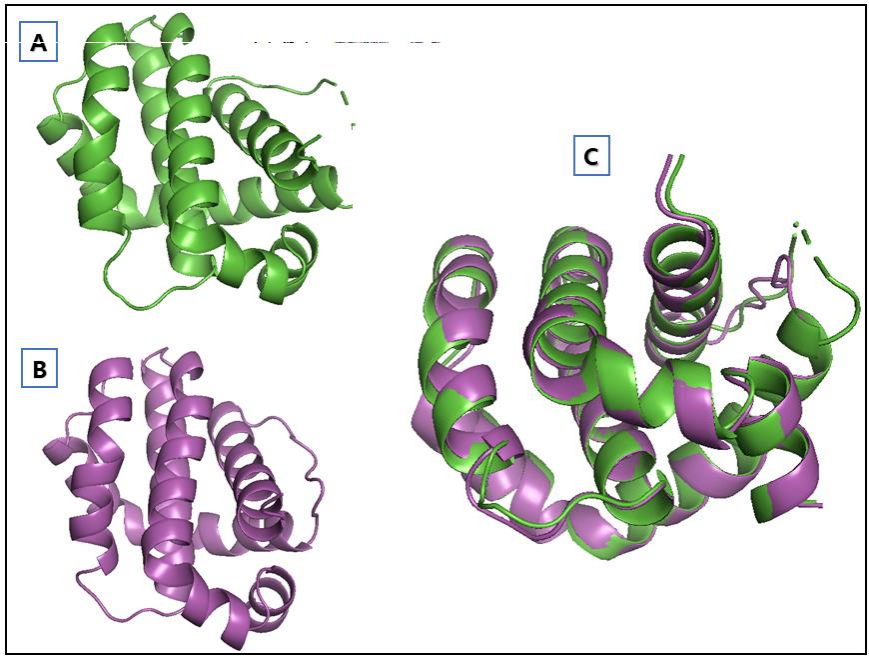
Refinement of the target protein 3 D structure. Originally retrieved structure from PDB (A), modelled structure (B), and alignment of original and modelled structure (C).

We performed a computational screening of 3,402 compounds with preclinical and clinical activity against the active sites of MCL-1 protein structures, prompted by the numerous success narratives of drug repurposing in cancer treatment. This screening sought to identify prospective candidates for cancer treatment repurposing. Due to distortions present in the retrieved 3D structure from the PDB, we employed the SWISS-MODEL tool to model the structure. Following the completion of the 3D structure modeling, we conducted a comparison by aligning the modeled structure with the PDB structure as the reference (Figure 1). The alignment revealed an RMSD value of 0.234 nm. As a positive control for this study, we chose 3-[3-(4-chloranyl-3,5-dimethyl-phenoxy) propyl]-~{N}-(phenylsulfonyl)-1~{H}-indole-2-carboxamide (5X2), gossypol, and venetoclax. 5X2 co-crystallized with the PDB structure, which is reported to possess a potent inhibitor of MCL-1 with an IC50 value of 400nM [15]. Gossypol [19] and venetoclax [20] are well-known BCL2/MCL-1 inhibitors.

The structure-based screening revealed that several compounds exhibited binding patterns similar to, and even superior to, the positive controls. Based on the analysis of binding affinity, the selected structure of MCL-1, specifically 5X2, displayed an affinity of -8.9 kcal/mol for the inbound ligand. Other controls such as Venetoclax exhibited -8.1 kcal/mol affinities, while Gossypol showed -7.7 kcal/mol affinity. By applying a cutoff based on the binding energy (BE) of 5X2, a total of 16 compounds exhibiting better BE are listed in Table 1. In addition, we estimated the physicochemical and drug likeness properties of these selected compounds to gain further insights. Although toxicity and ADME analysis are not typically required in drug repurposing approaches, we focused on predicting the general physicochemical and drug likeness properties of the compounds (Table 2).

**Figure 2:**
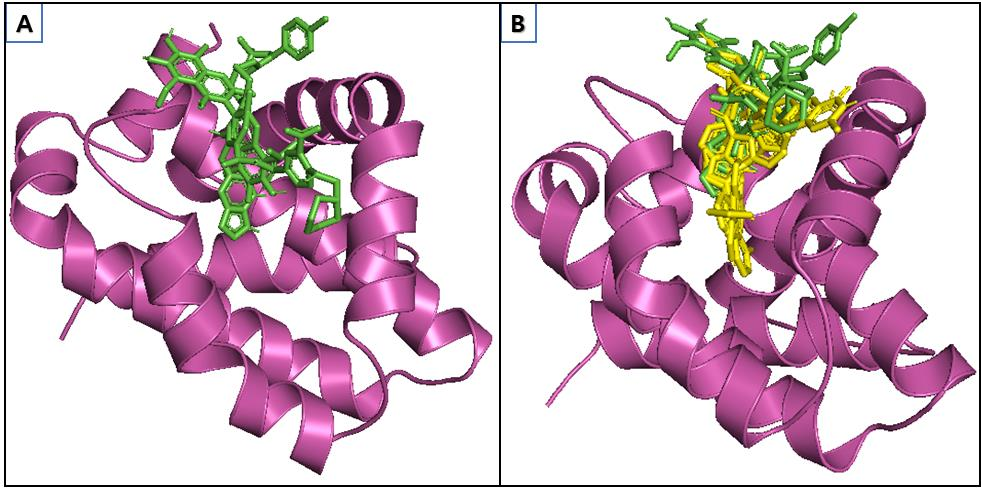
Superimpose visualization of (A) control compounds (5X2, Venetoclax, and Gossypol), and (B) hits (OSU-03012, Raltitrexed, Ostarine (MK-2866), Dovitinib (TKI-258), and Varespladib (LY315920)) in the MCL-1 active pocket.

**Figure 3:**
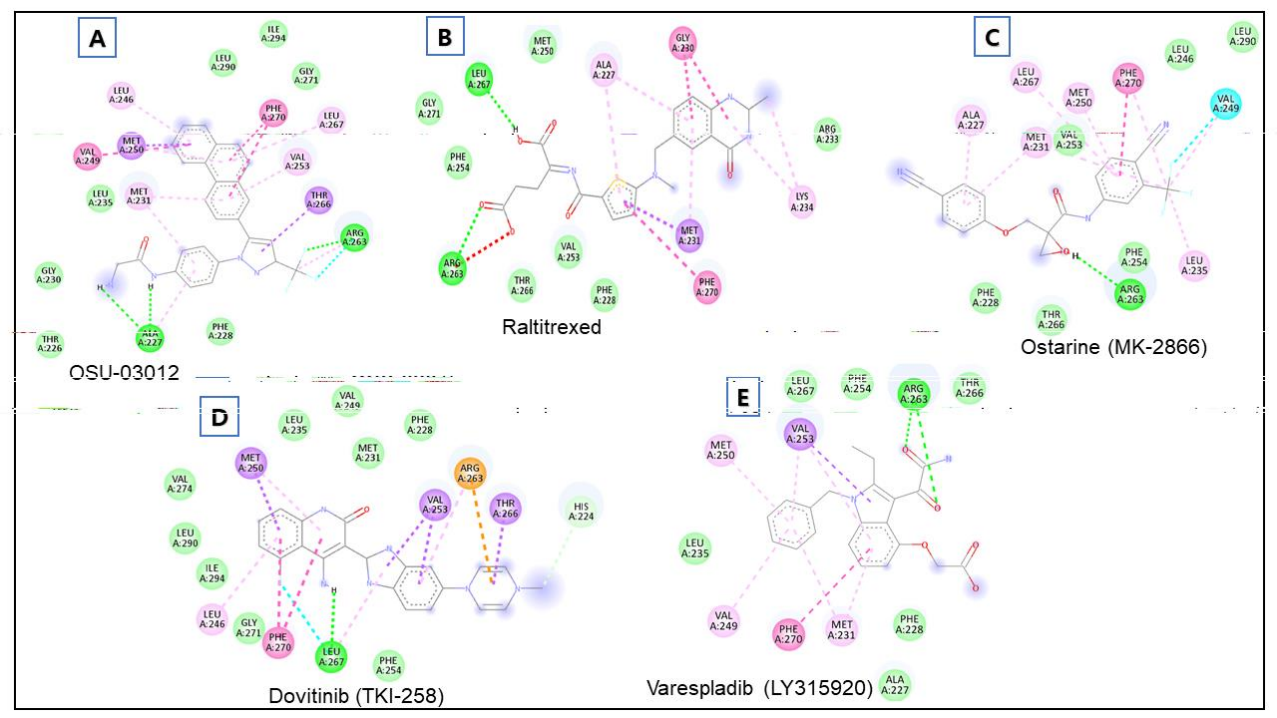
Interacting residues of MCL-1 protein with OSU-03012, Raltitrexed, Ostarine (MK-2866), Dovitinib (TKI-258), and Varespladib (LY315920).

Visual inspection and binding affinity analysis revealed that the compounds OSU-03012, Raltitrexed, Ostarine (MK-2866), Dovitinib (TKI-258), and Varespladib (LY315920) had strong binding for the MCL-1 protein (Figure 2). OSU-03012 was found to interact with Ala227, Thr226, Gly230, Met231, Leu235, Met250, Val249, Leu246, Leu290, Phe270, Ile294, Gly271, Leu267, Val253, Thr266, Arg263, and Phe228 residues of MCL-1. The Ala227 and Arg263 residues formed H-bond with OSU-03012 (Figure 3A). Raltitrexed interacted with Arg263, Phe254, Gly271, Leu267, Met250, Ala227, Gly230, Arg233, Lys234, Met231, Phe270, Phe228, Val253, and Thr266 residues of MCL-1. The Arg263, and Leu267 residues formed H-bond with Raltitrexed (Figure 3B). Ostarine (MK-2866) was found to interact with Arg263, Thr266, Phe228, Phe254, Leu235, Val249, Leu290, Leu246, Phe270, Met250, Val253, Met231, Leu267, and Ala227 residues of MCL-1. The Arg263 residue formed H-bond with Ostarine (MK-2866) (Figure 3C). Dovitinib (TKI-258) interacted with Leu267, Phe270, Gly271, Leu246, Ile294, Leu290, Val274, Met250, Leu235, Val249, Met231, Phe228, Arg263, Val253, Thr266, His224, and Phe254 residues of MCL-1. The Leu267 residue H-bonded with Dovitinib (TKI-258) (Figure 3D), Further, Varespladib (LY315920) was found to interact with Ala227, Phe228, Met231, Phe270, Val249, Leu235, Met250, Val253, Leu267, Phe254, Arg263, and Thr266 residues of MCL-1. The Arg263 residue formed H-bond with Varespladib (LY315920) (Figure 3E).

**Figure 4:**
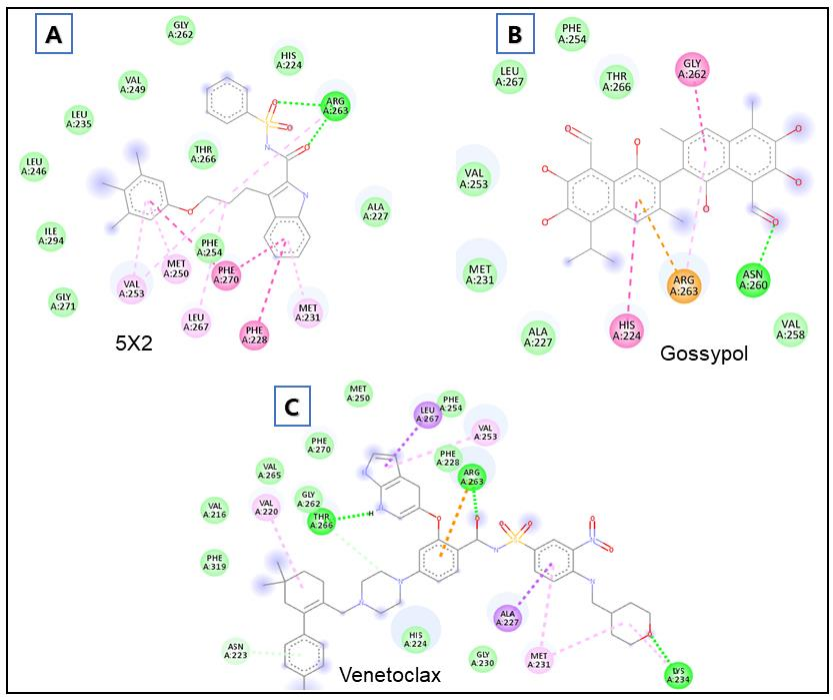
Interacting residues of MCL-1 protein with control compounds (5X2, Venetoclax, and Gossypol).

The interaction analysis for control compounds (5X2, Venetoclax, and Gossypol) was also performed. The cocrystal inhibitor (5X2) was found to bind with Gly271, Ile294, Leu246, Leu235, Val249, Gly262, His224, Arg263, Ala227, Thr266, Phe254, Met231, Phe228, Phe270, Leu267, Met250, and Val253 residues of MCL-1 (Figure 4A). Gossypol interacted with His224, Ala227, Met231, Val253, Leu267, Phe254, Thr266, Gly262, Val258, Asn260, and Arg263 residues of MCL-1 (Figure 4B). Further, Venetoclax was found to interact with Asn223, Phe319, Val216, Val220, Val265, Thr266, Gly262, Phe270, Met250, Leu267, Phe254, Val253, Phe228, Arg263, Lys234, Met231, Ala227, Gly230, and His224 residues of MCL-1 (Figure 4C). Remarkably, the hits (OSU-03012, Raltitrexed, Ostarine (MK-2866), Dovitinib (TKI-258), and Varespladib (LY315920)) and control compounds share several amino acid residues that engage in interactions with MCL-1.

Higher negative BE for ligand-protein complexes indicates stronger ligand binding to protein catalytic pocket and predicts low dissociation rates [21, 22, 23, 24, 25]. Interestingly, the hits (OSU-03012, Raltitrexed, Ostarine (MK-2866), Dovitinib (TKI-258), and Varespladib (LY315920)) have higher BEs than the control, indication that they have strong binding with the MCL-1 protein.

## Conclusion:

MCL-1 overexpression is common in various cancer types, including BC. In this study, the 3402 chemicals with established therapeutic activity against various diseases were screened against the MCL-1 protein. OSU-03012, Raltitrexed, Ostarine (MK-2866), Dovitinib (TKI-258), and Varespladib (LY315920) strongly bind to MCL-1 protein and interact with its key amino acid residues. These compounds have several amino acid residue interactions in common with the control compounds. These compounds could be used as MCL-1 inhibitors in the treatment of BC, however, further experimental validation is required.

## Author contributions:

AA, SA, MAK and QA: designed, data analysis and first draft the manuscript; AHA, AAA and MIS: literature survey and editing of THE manuscript; MHG and HMA, data collection and edit the manuscript. All authors read and approved the final version.

## Figures and Tables

**Table 1:** Best screened compounds with their respective binding affinity values.

**S.No**.	**Compounds name**	**Binding affinity/energy (kcal/mol)**
1.	Olaparib	-10.5
2.	Aprepitant	-10.3
3.	OSU-03012	-10.3
4.	Danoprevir	-10
5.	Regorafenib	-9.9
6.	Raltitrexed	-9.9
7.	Varespladib (LY315920)	-9.9
8.	Dovitinib (TKI-258)	-9.8
9.	Ostarine (MK-2866)	-9.8
10.	cyc116	-9.6
11.	Brivanib	-9.5
12.	CP-724,714	-9.2
13.	zm447439	-9.2
14.	MK-2206_dihydrochloride	-9.1
15.	SRT1720	-9.1
16.	bicalutamide	-9
17.	5X2 (control)	-8.9

**Table 2:** Physicochemical and Drug likeness properties prediction of selected compounds.

**Compounds name**	**cLogP**	**cLogS**	**HA**	**HD**	**Drug likeness**	**Mut**	**Tum**	**RE**	**Irr**	**PSA**
Olaparib	3.1669	-4.454	7	1	8.3247	X	X	X	X	82.08
Aprepitant	3.9633	-5.092	7	2	-1.3949	X	X	X	X	75.19
OSU-03012	4.2489	-7.054	5	2	-7.9989	high	high	X	X	72.94
Danoprevir	3.2878	-6.545	14	3	-58.243	X	X	X	X	188.9
Regorafenib	4.2436	-7.003	7	3	-5.1185	X	X	X	X	92.35
Raltitrexed	0.7203	-3.727	10	4	-5.3459	X	X	X	X	176.64
Dovitinib	0.7906	-2.392	7	3	7.3504	X	X	X	X	90.28
Varespladib (LY315920)	1.2599	-3.149	7	2	-0.75242	X	X	X	X	111.62
MK-2866	2.7147	-5.212	6	2	-7.8342	X	X	low	X	106.14
CYC116	3.0005	-4.54	7	2	2.8101	high	high	X	X	117.43
Brivanib	2.1159	-5.741	7	2	-3.4989	X	X	X	X	84.67
CP-724714	4.1071	-6.726	8	2	1.4469	X	X	X	X	98.26
ZM-447439	4.4258	-5.524	9	2	2.7563	high	high	X	X	97.84
MK-2206 dihydrochloride	3.8716	-7.24	6	2	2.4505	X	X	X	X	83.61
SRT1720 HCl	2.6245	-2.956	8	2	4.3964	X	X	X	low	115.69
Bicalutamide	2.1426	-5.084	6	2	-11.827	X	X	low	X	115.64
HA: H-Acceptors
HB: H-Donors
Mut: Mutagenic
Tum: Tumorigenic
RE: Reproductive Effective
Irr: Irritant
PSA: Polar Surface Area
